# Organization and Plasticity of Sodium Channel Expression in the Mouse Olfactory and Vomeronasal Epithelia

**DOI:** 10.3389/fnana.2017.00028

**Published:** 2017-04-03

**Authors:** Florian Bolz, Stephanie Kasper, Bernd Bufe, Frank Zufall, Martina Pyrski

**Affiliations:** Center for Integrative Physiology and Molecular Medicine, Saarland UniversityHomburg, Germany

**Keywords:** voltage-gated sodium channels, olfactory epithelium, vomeronasal organ, development, regeneration

## Abstract

To understand the molecular basis of neuronal excitation in the mammalian olfactory system, we conducted a systematic analysis of the organization of voltage-gated sodium (Na_v_) channel subunits in the main olfactory epithelium (MOE) and vomeronasal organ (VNO) of adult mice. We also analyzed changes in Na_v_ channel expression during development in these two systems and during regeneration of the MOE. Quantitative PCR shows that Na_v_1.7 is the predominant isoform in both adult MOE and VNO. We detected pronounced immunoreactivity for Na_v_1.7 and Na_v_1.3 in axons of olfactory and vomeronasal sensory neurons (VSNs). Analysis of Na_v_1.2 and Na_v_1.6 revealed an unexpected subsystem-specific distribution. In the MOE, these Na_v_ channels are absent from olfactory sensory neurons (OSNs) but present in non-neuronal olfactory cell types. In the VNO, Na_v_1.2 and Na_v_1.6 are confined to VSNs, with Na_v_1.2-immunoreactive somata solely present in the basal layer of the VNO. The subcellular localization of Na_v_1.3 and Na_v_1.7 in OSNs can change dramatically during periods of heightened plasticity in the MOE. During the first weeks of development and during regeneration of the olfactory epithelium following chemical lesion, expression of Na_v_1.3 and Na_v_1.7 is transiently enhanced in the somata of mature OSNs. Our results demonstrate a highly complex organization of Na_v_ channel expression in the mouse olfactory system, with specific commonalities but also differences between the MOE and the VNO. On the basis of their subcellular localization, Na_v_1.3 and Na_v_1.7 should play major roles in action potential propagation in both MOE and VNO, whereas Na_v_1.2 and Na_v_1.6 are specific to the function of VSNs. The plasticity of Na_v_ channel expression in OSNs during early development and recovery from injury could reflect important physiological requirements in a variety of activity-dependent mechanisms.

## Introduction

Olfactory information processing and olfactory performance relies on the detection and transmission of peripheral olfactory information to the brain. This process begins within specialized bipolar neurons, the olfactory sensory neurons (OSNs), located in the main olfactory epithelium (MOE). The cilia and distal dendrites of an OSN perform the primary chemo-electrical signal transduction process to generate graded receptor potentials in response to odor detection (Firestein, 2001; Kleene, 2008; Pifferi et al., 2010). These electrical signals at the input level of an OSN are subsequently transformed into action potential sequences passing along the OSN axons to the olfactory bulb of the forebrain. In the glomerular neuropil of the olfactory bulb, OSN axon terminals synapse onto second order neurons, the mitral and tufted cells that convey olfactory information to higher brain centers (Shepherd et al., 2004; Wachowiak and Shipley, 2006). During the past 25 years, we have obtained detailed information on the molecular mechanisms underlying primary olfactory signal transduction in mammalian OSNs, but very little is still known about the molecular details underlying the initiation and propagation of action potentials in these sensory neurons. A comprehensive analysis of the function of voltage-activated ion channels in OSNs, specifically the voltage-activated sodium (Na_v_) channels, will be required for a molecular basis of neuronal excitation in the mammalian olfactory system, and for understanding the causes of heritable disorders underlying olfactory dysfunction in humans.

The sodium channel Na_v_1.7 (encoded by the gene *Scn9a*) plays an essential role in mammalian olfaction: loss-of-function mutations in this gene cause a loss of the sense of smell (congenital general anosmia) in both mice and humans (Weiss et al., 2011). However, Na_v_1.7-deficient mouse OSNs can still generate action potentials in response to odorants, although the cells fail to propagate a signal to the target neurons in the olfactory bulb (Weiss et al., 2011). Therefore, we reasoned that other Na_v_ channel isoforms must ultimately play additional roles in OSN excitation. Nine structurally related Na_v_ channel α-subunits (Na_v_1.1–Na_v_1.9; Goldin et al., 2000; Catterall et al., 2005) are known to exist in mammals and differ in their tissue specificity, biophysical properties, and temporal expression during development and regeneration (Waxman et al., 1994; Kim et al., 2002; Dib-Hajj et al., 2010). Consistent with the cellular and behavioral phenotypes of mice harboring a conditional knockout mutation in *Scn9a* (Weiss et al., 2011), several additional investigations have provided evidence that Na_v_1.7 is not the only sodium channel expressed in the peripheral olfactory system, but that other isoforms are also present and could perform specific roles in excitation. In the MOE, several different Na_v_ isoforms have been identified in mouse OSNs by expression profiling (Sammeta et al., 2007), RT-PCR (Ahn et al., 2011; Weiss et al., 2011; Frenz et al., 2014), and deep RNA sequencing (Ibarra-Soria et al., 2014). Only recently, Na_v_1.7, Na_v_1.3 (Weiss et al., 2011), and Na_v_1.5 (Frenz et al., 2014) were identified in mouse OSNs in addition to Na_v_1.7 in rat OSNs (Ahn et al., 2011) using immunohistochemistry. In the vomeronasal organ (VNO), very little information is available on Na_v_ channel expression in vomeronasal sensory neurons (VSNs). Although multiple isoforms have been identified by RT-PCR (Fieni et al., 2003) and deep RNA sequencing (Ibarra-Soria et al., 2014), only a single study (Rupasinghe et al., 2012) localized Na_v_1.7 protein to the nerve and glomerular layers of the accessory olfactory bulb. What is still missing, however, is a systematic analysis of the organization of multiple Na_v_ channel subunits in olfactory peripheral tissues. Such investigations are also required to address the critical question whether sensory neurons of the two major olfactory organs in mice—OSNs in the MOE and VSNs in the VNO—each may have evolved distinct mechanisms for neuronal excitation, or whether they employ the same Na_v_ channels for action potential generation and conduction despite the fact these olfactory subsystems have evolved strikingly distinct mechanisms for primary signal transduction (Munger et al., 2009; Zufall and Munger, 2016).

Here, we analyze the cellular and subcellular distribution of different Na_v_ channel subtypes in the peripheral olfactory system of mice during adulthood and development (MOE and VNO), and during regeneration following chemical lesions (MOE). Our results show that Na_v_1.7 is the most abundant subtype not only in the MOE but also in the VNO. Furthermore, our immunohistochemical evidence suggests that both olfactory subsystems may employ Na_v_1.3 and Na_v_1.7 for axonal propagation of action potentials. We also find that these two channels likely play a major role in the developing MOE as they undergo specific changes in subcellular localization in OSNs during the first weeks of life. Finally, we demonstrate that Na_v_1.2 and Na_v_1.6 locate specifically to subpopulations of sensory neurons in the VNO.

## Materials and Methods

### Animals

All procedures were approved by the Institutional Animal Care and Use Committee of Saarland University and were in full accordance with the laws for animal experiments of the German government. Experiments were performed on mouse tissues derived from mice at different ages and of both sexes. For the developmental study, we used mice at embryonic day 18 (E18), postnatal day 2 (P2), P7, P14 and P21. Results from adult mice were obtained at 6–8 weeks of age. Ages of mice used for the regeneration study are as specified in “Triton X Lesioning of the Main Olfactory Epithelium” Section. We used wild type mice (C57BL/6J, denoted as B6), cNa_v_1.7 mice (Weiss et al., 2011) and OMP–GFP^+/−^ mice (B6; 129P2–Omp^tm3Mom^/MomJ, The Jackson Laboratory; stock# 006667), heterozygous for both OMP (olfactory marker protein) and GFP (green fluorescent protein; Potter et al., 2001). Mice were housed in micro-isolator cages on a 12:12-h light/dark cycle with water and food available *ad libitum*.

### RNA Extraction and Quantitative Real Time RT-PCR

Olfactory mucosa and VNO were obtained from 6 to 8-week old B6 mice. Total RNA was isolated using the InnuPREP RNA isolation kit (Analytik Jena). Quality was assessed by gel electrophoresis and photometric measurements. cDNA was synthesized from 0.5 μg of total RNA using the Smart cDNA Synthesis Kit (Clontech) and Superscript II reverse transcriptase (Invitrogen). Quantitative PCR for the different mouse Na_v_ subunits was done on a My-iQ-cycler (Bio-Rad) using iQ^TM^ SYBR^®^ Green Supermix (Bio-Rad) according to the manufacturers’ recommendations. Forward and reverse gene specific primers used were Na_v_1.1 (AGCCTGGTAGAACTTGGCCTTGC and TGCCAACCACGGCAAAAATAAAG), Na_v_1.2 (TGGGATCTTCACCGCAGAAATG and TGGGCCAGGATTTTGCCAAC), Na_v_1.3 (AGCTTGGCCTGGCAAACGTG and ATGCCGACCACGGCAAAAATG), Na_v_1.5 (ACAGCCGAGTTTGAGGAGATGC and CGCTGATTCGGTGCCTCA), Na_v_1.6 (ACGCCACAATTCGAACATGTCC and CCTGGCTGATCTTACAGACGCA), Na_v_1.7 (ACGGATGAATTCAAAAATGTACTTGCAG and GTTCTCGTTGATCTTGCAAACACA). PCR conditions were: 95°C for 3 min initial denaturation, followed by 42 cycles of 95°C for 30 s, 64°C for 20 s, 72°C for 30 s. Triplicate reactions were performed on 96-well plates and analyzed with the iQ5 Software (Bio-Rad). Quality controls for PCR conditions, linearity of the amplification reaction and RNA isolation were assessed according to MIQE guidelines (Bustin et al., 2009). In addition, the specificity of PCR products was confirmed by gel electrophoresis and by direct DNA sequencing of the PCR products. For copy number calculation calibration curves for each primer set with defined amounts of start copies diluted in tRNA containing reaction buffer were used.

### Olfactory Tissue Preparation

Perfusion of mice and tissue preparation followed previously described methods (Weiss et al., 2011). In brief, mice were sacrificed by anesthesia (165 mg/kg body weight ketamine (Pharmacia GmbH, Berlin, Germany) and 11 mg/kg body weight xylazine (Bayer Health Care, Leverkusen, Germany)) and subjected to transcardial perfusion using phosphate buffered saline (PBS) followed by perfusion with 4% (w/v) paraformaldehyde in PBS as fixative. Two day-old mice and mice at embryonic day 18 (E18) were decapitated and fixated by immersion in 4% PFA for 24 h instead of transcardial perfusion. E18 embryos were dissected from anesthetized, time-pregnant females. Following fixation, olfactory tissues were incubated in 30% sucrose in PBS at 4°C for 2 days, embedded in O.C.T. (Tissue-Tek), and snap-frozen in a dry ice/2-methylbutane bath. Frozen tissue sections (12 μm) were collected on a cryostat (HM525; Microm, Walldorf, Germany), thaw-mounted onto glass slides (Superfrost Plus, Polysciences), and stored at −80°C.

### Triton X Lesioning of the Main Olfactory Epithelium

For peripheral deafferentation, young adult OMP-GFP mice or B6 mice (6–8 weeks) received unilateral intranasal irrigation with 100 μl of a 0.7% Triton X-100 solution prepared in phosphate-buffered saline (PBS), pH 7.4. Mice were two-hand scruff restrained to prevent head movement to inject 100 μl solution into the left nasal cavity using a 21-gauche, blunt-end needle (Braun, Melsungen) attached to a 1 ml syringe. Following intranasal treatment, mice were monitored to ensure complete recovery and allowed to survive for 1, 2, 4, 6, 8 and 10 weeks (*n* = 2 mice each) before subjected to transcardial perfusion and olfactory tissue preparation as detailed above.

### Immunohistochemistry

All procedures were conducted at room temperature (20°C), only incubation of tissue sections with primary antibodies was at 4°C. The following primary antibodies and control peptides were used: Na_v_1.2 (1:500, rabbit polyclonal AB5206, control peptide ASAESRDFSGAGGIGVFSE, Millipore), Na_v_1.3 (1:500, rabbit polyclonal AB5208, control peptide HLEGNHRADGDRFP, Millipore) or Na_v_1.3 (1:500, goat polyclonal sc-22202, St. Cruz), Na_v_1.6 (1:500, rabbit polyclonal ASC-009, control peptide CIANHTGVDIHRNGDFQKNG, Alomone), Na_v_1.7 (1:500, rabbit polyclonal AB5390, control peptide EFTSIGRSRIMGLSE, Millipore), OMP (1:3000, goat polyclonal; gift of F. Margolis, University of Maryland, Baltimore, MD, USA), V2R2 (1:5000, rabbit polyclonal; provided by R. Tirindelli, University of Parma, Parma, Italy), GAP43 (1:2000, mouse monoclonal MAP347, Millipore).

Expression of Na_v_ channel isoforms was detected by tyramid signal amplification according to the manufacturer’s protocol (TSA-Biotin System, Perkin Elmer). In brief, coronal cryosections (12–14 μm) of the VNO and MOE were brought to room temperature, rinsed in TN-buffer (100 mM Tris, 150 mM NaCl, pH 7.5), incubated in 3% H_2_O_2_ for 10 min, washed in TN-buffer, and incubated for 2 h in blocking solution containing 4% normal horse serum (Vector Laboratories) and 0.3% Triton X-100 (Sigma) prepared in TN. Then, sections were sequentially incubated in primary antibody diluted in blocking solution for 18–24 h, in biotinylated donkey-anti-rabbit antibody (1:400, #711-065-152, Jackson Immuno Research) or in biotinylated horse-anti-goat antibody (1:400, BA-9500, Vector Laboratories) for 1 h, in streptavidin-HRP (1:100, TSA-Biotin System, Perkin Elmer) for 30 min, in biotinylated tryamid (1:100, TSA-Biotin System, Perkin Elmer) for 10 min, and in Alexa 546-conjugated streptavidin (1:200; S-11225, Invitrogen) or in Alexa 633-conjugated streptavidin (1:200; S-21375 Invitrogen). Nuclei were counterstained with Hoechst 33342 nuclear dye (Invitrogen, 1:10,000) for 10 min and sections were cover slipped in fluorescence mounting medium (DAKO). For the colocalization of Na_v_1.7 with Na_v_1.3, we performed two sequential TSA amplifications using the Na_v_1.7 antibody made in rabbit followed by the Na_v_1.3 antibody made in goat using the appropriate biotinylated secondary antibodies (see above). In between the two TSA protocols Biotin blocking was performed using the Avidin/Biotin blocking kit (Vector Laboratories). GAP43, OMP and V2R2 were detected by indirect immunofluorescence after TSA-amplification of Na_v_ channels using the secondary antibodies Alexa-Fluor 488 conjugated donkey-anti-goat or Alexa-Fluor 647 conjugated donkey-anti-mouse (all 1:1000, Invitrogen). For V2R2 detection, unbound rabbit IgG epitopes from the previous TSA protocol were blocked for 1 h with donkey-anti-rabbit Fab-fragments (1:50, Biomol, Rockland, ME, USA). The specificity of the immunostainings was verified by several types of control experiments: (i) omitting primary antibody; (ii) incubation peptide pre-adsorpt Na_v_ antisera (5-fold excess of the cognate immunization peptide); and (iii) applying Na_v_ antibodies to olfactory tissue derived from cNa_v_1.7 knockout mice (Weiss et al., 2011).

### Microscopy and Image Assembly

Fluorescence images were acquired on a BX61 epifluorescence microscope attached to a DP71 camera (Olympus) or on a LSM 710/ConfoCor-3 confocal microscope (Zeiss). Confocal images are Z-stacks presented as maximum intensity projections of 10–20 confocal sections, each 0.4 μm thick. Images were assembled and minimally adjusted in contrast and brightness using Photoshop Elements 10 (Adobe Photoshop).

## Results

To understand the molecular basis of neuronal excitation in the mammalian olfactory system, we used a combination of quantitative real time RT-PCR (qPCR) and immunohistochemistry and analyzed the organization and plasticity of Na_v_ channel α-subunit expression in the peripheral olfactory system of mice. We focused on the MOE and the VNO which represent the two major sensory substructures of the mouse olfactory system (Munger et al., 2009). We describe the neural architecture and subcellular distribution of the major Na_v_ channel subunits that we could identify in these sensory epithelia during adulthood and development of the MOE and VNO, and during regeneration of the MOE following chemical epithelial ablation.

### qPCR Reveals Na_v_1.7 as the Most Abundant Na_v_ Channel in both MOE and VNO

**Figure 1:**
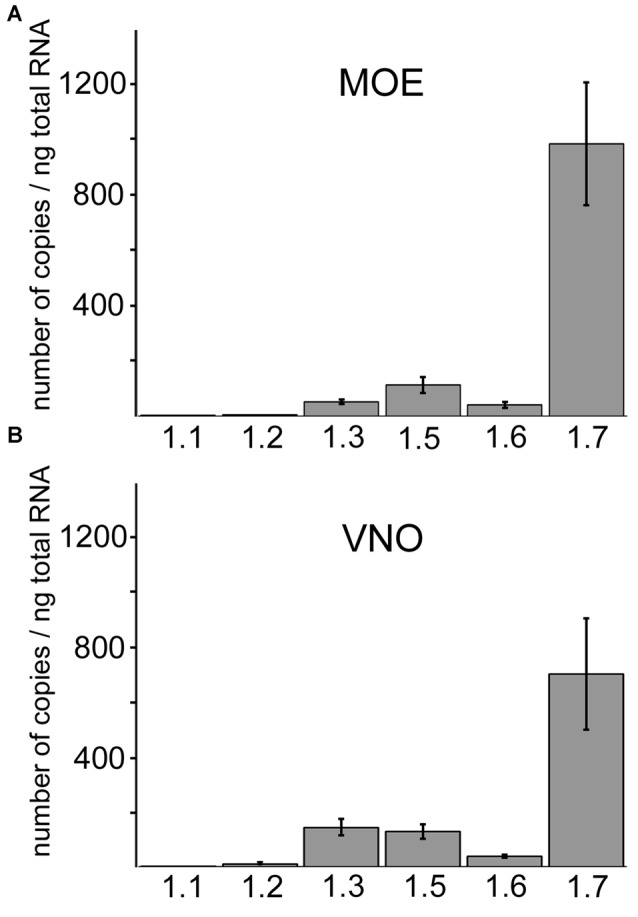
**Quantitative RT-PCR of different Na_v_ isoforms in main olfactory epithelium (MOE) and vomeronasal organ (VNO).** Comparison of Na_v_ channel mRNA frequency in the **(A)** MOE and **(B)** VNO using quantitative RT-PCR. Na_v_1.7 is the most abundant isoform in both subsystems and displays on average at least 5-fold higher copy numbers than other Na_v_ isoforms. The column diagrams show mean copy numbers ± SD from two to four independent experiments, each carried out as triplicates using total RNA of adult C57/B6 mice (*Y*-axis). Na_v_ channel isoforms (*X*-axis). **(A)** Copy numbers per ng total RNA in the MOE for Na_v_1.1 (2.6 ± 0.72), Na_v_1.2 (4.99 ± 0.62), Na_v_1.3 (51.80 ± 7.75), Na_v_1.5 (113.17 ± 27.35), Na_v_1.6 (41.19 ± 10.65), Na_v_1.7 (983.44 ± 221.34). **(B)** Copy numbers per ng total RNA in the VNO for Na_v_1.1 (0.80 ± 0.42), Na_v_1.2 (10.16 ± 2.10), Na_v_1.3 (142.70 ± 29.05), Na_v_1.5 (128.13 ± 25.56), Na_v_1.6 (38.17 ± 5.39), Na_v_1.7 (701.06 ± 203.50).

Sensory neurons of MOE and VNO employ distinct primary signal transduction mechanisms but it is unclear whether these two olfactory subsystems have also evolved distinct molecular mechanisms for action potential generation and propagation or whether they employ the same mechanisms. To assess this question, we first compared the expression profiles of different Na_v_ isoforms in the two subsystems. We used total RNA preparations from MOE and VNO of wild type B6 mice and conducted qPCR analysis using gene-specific primers for six members of the Na_v_ family: Na_v_1.1 (*Scn1a*), Na_v_1.2 (*Scn2a*), Na_v_1.3 (*Scn3a*), Na_v_1.5 (*Scn5a*), Na_v_1.6 (*Scn8a*), and Na_v_1.7 (*Scn9a*). Quantitative analyses revealed that overall relative abundances of the different Na_v_ channel isoforms in whole MOE and VNO were surprisingly similar (Figure 1). In both tissues, mRNA encoding the Na_v_1.7 α-subunit represented by far the most abundant isoform. We also detected mRNA encoding the isoforms Na_v_1.5, Na_v_1.3, Na_v_1.6, Na_v_1.2 and Na_v_1.1 in both tissues (see legend of Figure 1 for mRNA copy numbers).

### In the MOE Na_v_1.3 and Na_v_1.7 Represent the Predominant Na_v_ Channel Isoforms

**Figure 2:**
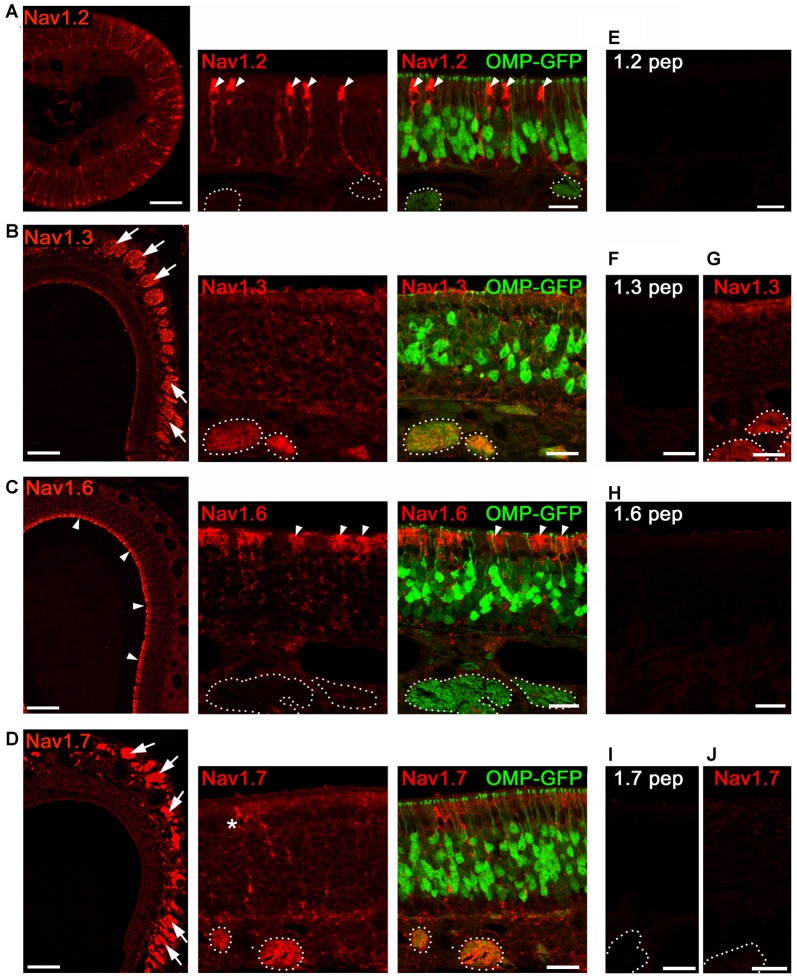
**Analysis of Na_v_ channel expression in MOE of adult mice.** Confocal images showing immunoreactivity (red) for **(A)** Na_v_1.2, **(B)** Na_v_1.3, **(C)** Na_v_1.6, and **(D)** Na_v_1.7. We used coronal MOE cryosections (12 μm) of adult OMP-GFP mice (left, overviews; right, magnifications). Endogenous GFP (green) is located to mature, OMP^+^ OSNs as shown in the magnifications at the right. **(A)** Na_v_1.2 staining is restricted to microvillar cells (arrowheads) but absent from OSNs or axon bundles (dotted circles) identified by OMP-GFP labeling. **(B)** Robust Na_v_1.3 staining is present in axon bundles (arrows, left) that colocalize with OMP-GFP (dotted circles, right). **(C)** Na_v_1.6 labeling of the MOE surface (left, arrow heads) corresponds to sustentacular cells (right). OSNs and axon bundles (dotted line) lack Na_v_1.6 staining. **(D)** Na_v_1.7 immunoreactivity is profound in axon bundles, and occasionally found in microvillar cells (asterisk). **(E,F,H,I)** Blocking peptide control experiments lack immunoreactivity. **(G)** Na_v_1.3 immunoreactivity is present in cNa_v_1.7^−/−^ mice. **(J)** Na_v_1.7 immunoreactivity is absent in cNa_v_1.7^−/−^ mice. Images for each Na_v_ channel immunostaining are representatives of *n* ≥ 3 mice and *n* = 20 sections per mouse. Scale bars overviews 100 μm, and magnifications 20 μm.

In addition to OSNs that mediate the sense of smell, the MOE encompasses non-neuronal supporting and microvillous cells, as well as dividing stem cells that form the olfactory mucosa (Farbman, 1992). To verify that the Na_v_ isoforms we identified by qPCR localize to OSNs, we performed immunohistochemistry for the isoforms Na_v_1.2, Na_v_1.3, Na_v_1.6 and Na_v_1.7 in the MOE of adult mice (Figure 2) using previously established antibodies (Sage et al., 2007; Gao et al., 2009; Weiss et al., 2011). We did not investigate the distribution of Na_v_1.5 in OSNs as this has already been reported by Frenz et al. (2014). Olfactory tissue sections from B6 mice or from heterozygous OMP-GFP mice were subjected to immunohistochemistry using tyramid signal amplification. OMP-GFP mice express the reporter GFP (green fluorescence protein) under control of the promotor of OMP (olfactory marker protein). Thus, mature OSNs can be readily identified by their endogenous GFP fluorescence in these mice.

Of the four candidates analyzed in adult MOE, we identified robust immunostaining for Na_v_1.3 and Na_v_1.7 in OSN axon bundles (Figures 2B,D) located in the lamina propria underlying the MOE. Axon bundles were identified through endogenous GFP fluorescence in OMP-GFP mice (Figures 2B,D). OSN somata showed relatively little Na_v_1.3 and Na_v_1.7 immunostaining which was homogenously distributed throughout the depth of the MOE. OSN dendrites or dendritic endings (knobs) lacked immunoreactivity altogether (for summary see Figure 7).

By contrast, staining for Na_v_1.2 and Na_v_1.6 was absent from OSNs (Figures 2A,C), but we observed robust staining for Na_v_1.2 in a discrete population of non-neuronal microvillar cells (Figure 2A). These cells can be identified by their specific morphology including a club-shaped soma that is positioned in the most apical MOE layer, extending a thick process towards the basal membrane (Elsaesser and Paysan, 2005). Consistent with a previous report (Frenz et al., 2014), we also noticed a subset of MOE microvillar cells that were labeled for Na_v_1.7 (Figures 2B,D). We observed Na_v_1.6 immunoreactivity in the apical cytosol of sustentacular cells, a non-neuronal cell type of the MOE with glia-like supportive function (Farbman, 1992).

As controls for antibody specificity, we omitted primary antibodies to control binding of secondary antibodies to the tissue. We also conducted antibody blocking experiments by preincubation of each antiserum with its cognate immunization peptide. In all cases, reactions were devoid of any signal for the Na_v_ channels investigated (Figures 2E,F,H,I). Furthermore, control experiments using tissue from cNa_v_1.7^−/−^ knockout mice, in which Na_v_1.7 has been deleted in all OMP-expressing cells (Weiss et al., 2011), lacked Na_v_1.7 immunoreactivity (Figure 2J). Moreover, immunoreactivity for Na_v_1.3 was not affected by the Na_v_1.7 deletion and we observed prominent Na_v_1.3 staining in axon bundles of cNa_v_1.7^−/−^ mice (Figure 2G).

### Na_v_1.2 and Na_v_1.6 Localize to VSN Somata in the VNO

**Figure 3:**
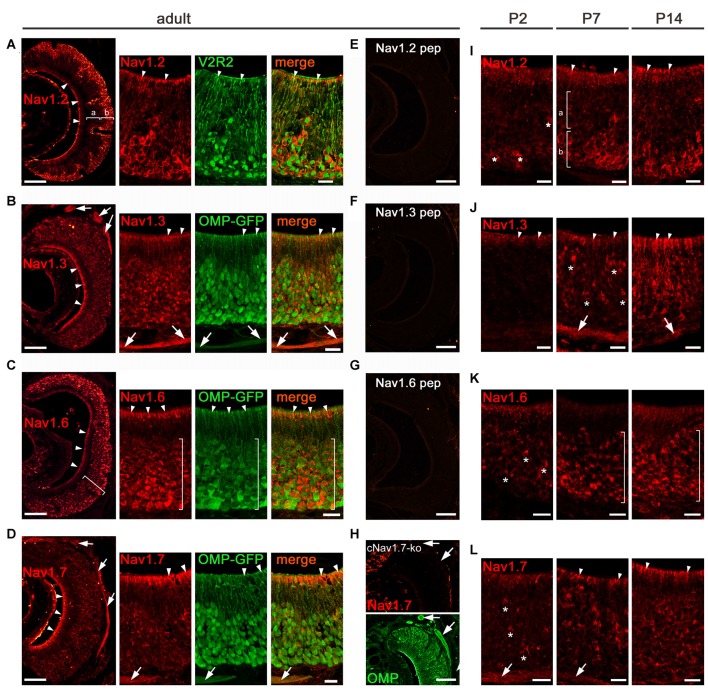
**Analysis of Na_v_ channel expression in VNO of adult and early postnatal mice. (A–H)** Confocal images showing immunoreactivity (red) observed for **(A)** Na_v_1.2, **(B)** Na_v_1.3, **(C)** Na_v_1.6, and **(D)** Na_v_1.7 in 12 μm coronal VNO cryosections of adult mice (left, overviews; right, magnifications). **(A)** Na_v_1.2 staining is present in VSN knobs (arrowheads), dendrites and somata of basal (b) VSNs but absent in apical (a) VSNs (overview left). The magnification at the right shows colocalization of Na_v_1.2 with V2R2 (green). **(B)** Na_v_1.3 staining is moderate in VSN knobs (arrowheads), dendrites and somata and robust in axon bundles as identified in OMP-GFP mice (green signal). **(C)** Na_v_1.6 is strong in knobs (arrowheads) and somata (bracket). The magnification and colocalization with OMP-GFP shows a decline in Na_v_1.6 intensity from basal to apical. **(D)** Na_v_1.7 staining is prominent in VSN knobs (arrowheads) and axon bundles (arrows). Peptide control experiments **(E,F,G)** lack immunoreactivity. **(H)** Na_v_1.7 staining (top) is absent in axon bundles (arrows) of cNa_v_1.7^−/−^ mice, visualized by OMP staining (bottom). **(I-L)** Immunoreactivity (red) for **(I)** Na_v_1.2, **(J)** Na_v_1.3, **(K)** Na_v_1.6, and **(L)** Na_v_1.7 at postnatal (P) day 2, P7, and P14. **(I)** Onset of Na_v_1.2 in P2 VSNs (asterisks). At P7 and P14, basal (b) confinement of Na_v_1.2 somata and labeled knobs (arrowheads; a). **(J)** Na_v_1.3 is visible in single somata (asterisks) and axon bundles (arrows) at P7, and shows diffuse staining of the apical VNO (arrowheads) starting at P2. **(K)** Onset of Na_v_1.6 in somata (asterisks) at P2 with increasing numbers at P7 and P14 throughout the VNO (brackets).** (L)** Na_v_1.7 in single VSN somata (asterisks) and axon bundles (arrows) starts at P2. The knob area is substantially stained from P7 onwards. Images are representatives of *n* ≥ 3 adult mice (*n* = 20 sections per mouse) and *n* = 2 juvenile mice each at P2, P7, and P14 (*n* ≥ 10 sections per mouse). Scale bars overviews (**A–H**, left) and **(E–H)** 100 μm; magnifications **(A–D)** and **(I–L)** 20 μm.

In the VNO, our qPCR experiments suggested that Na_v_1.7 is not the sole Na_v_ channel (Figure 1B). By performing immunohistochemistry analyses in VNO tissue sections from adult mice, we localized all four channel candidates—Na_v_1.2, Na_v_1.3, Na_v_1.6, and Na_v_1.7—to VSNs (for summary see Figure 7). Interestingly, we observed profound Na_v_1.2 staining in VSNs residing in the basal VNO layer (Figure 3A). Colocalization with an antibody against the vomeronasal receptor V2R2, specific for family C V2Rs that show expression in almost all basal VSNs (Martini et al., 2001), confirmed the confinement of Na_v_1.2 to basal VSNs (Figure 3A).

Na_v_1.2 labeling appeared to be primarily restricted to VSN somata, dendrites, and dendritic knobs (Figure 3A) as we did not detect Na_v_1.2 in VSN axon bundles leaving the VNO and forming the vomeronasal nerves (Figure 3A). Surprisingly, we observed a similar subcellular distribution for Na_v_1.6 that showed strong immunoreactivity in VSN somata and knobs whereas axon bundles were devoid of any staining (Figure 3C). In contrast to Na_v_1.2, we detected Na_v_1.6 staining in VSNs of both apical and basal VNO layers. This was confirmed in the VNO of OMP-GFP mice in which virtually all GFP^+^ VSN somata were also labeled for Na_v_1.6 (Figure 3C). However, the signal intensity for Na_v_1.6 in basal VSNs appeared stronger than that in apical VSNs. The subcellular localization of the two channel subtypes Na_v_1.2 and Na_v_1.6 to VSN somata makes them ideal candidates involved in the generation of action potentials in VSNs.

### Na_v_1.3 and Na_v_1.7 Localize to VSN Axons

Next, we analyzed the distribution of Na_v_1.3 and Na_v_1.7 in the adult vomeronasal sensory epithelium. We detected robust immunoreactivity for both channel subtypes in axon bundles forming the vomeronasal nerves, situated in the dorsomedial aspect of the VNO (Figures 3B,D). Furthermore, we found moderate Na_v_1.3 staining in VSN dendrites and dendritic knobs as well as in VSN somata (Figure 3B). For Na_v_1.7, in addition to the striking labeling of axon bundles, we observed substantial staining in VSN dendritic knobs whereas immunoreactivity was rather weak in VSN somata and dendrites (Figure 3D). VSN somata stained for Na_v_1.3 and Na_v_1.7 were present throughout the depth of the vomeronasal sensory epithelium.

As a control, we analyzed the VNO from cNa_v_1.7^−/−^ mice in which Na_v_1.7 has been conditionally deleted in all OMP-expressing cells including all VSNs (Weiss et al., 2011). Consistent with our results in the MOE, cNa_v_1.7^−/−^ mice lacked Na_v_1.7 staining in the VNO (Figure 3H). Furthermore, immunoreactivity was absent when omitting primary antibodies (not shown), as well as in peptide control reactions (Figures 3E–G).

Taken together, the results depicted in Figure 3 reveal characteristic, differential expression in distinct VSN compartments for the four Na_v_ channel subtypes investigated. Na_v_1.2 and Na_v_1.6 are predominantly expressed in VSN cell bodies. Na_v_1.3 and Na_v_1.7 are mainly, but not exclusively, expressed in VSN axons. Na_v_1.2 constitutes a novel marker for VSNs of the basal layer of the vomeronasal sensory epithelium and we predict it to play a specific function in these neurons.

### Na_v_ Channel Expression during Early Postnatal VNO Development

Changes in the subcellular distribution of Na_v_ channels during development could impact on the electrical activity and generation of nerve impulses produced by VSNs and OSNs during this time. We investigated the localization of the four Na_v_ channels at different developmental time points using immunohistochemistry, focusing first on the VNO. We analyzed VNO tissue sections derived from B6 or OMP-GFP mice on tissue sections of mice at postnatal day 2 (P2), P7, and P14 (Figures 3I–L). We found Na_v_1.2 and Na_v_1.6 immunoreactivity in VSN somata starting at P2 with increasing numbers of labeled neurons towards P14 (Figures 3I,K). Axon bundles were devoid of any staining in both cases. As seen in adult mice (Figure 3A), restriction of Na_v_1.2 stained somata to the basal VNO layer was already prominent at P7 (Figure 3I). Na_v_1.6 labeled somata, however, were found at all depths of the vomeronasal epithelium at P7 (Figure 3K), closely resembling the distribution in the adult VNO. Substantial Na_v_1.7 staining of individual VSN somata, knobs, and axon bundles was detectable at P2 (Figure 3L), while Na_v_1.3 staining in these compartments became evident at P7 (Figure 3J), with numbers of labeled VSNs increasing towards P14.

Thus, Na_v_ channel expression in the developing VNO is detectable in VSNs shortly after birth, increases with age, and the particular cellular and subcellular distribution pattern of each channel type is formed during the first 2 weeks of postnatal development.

### The Subcellular Distribution of Na_v_1.3 and Na_v_1.7 in OSNs is Developmentally Regulated

**Figure 4:**
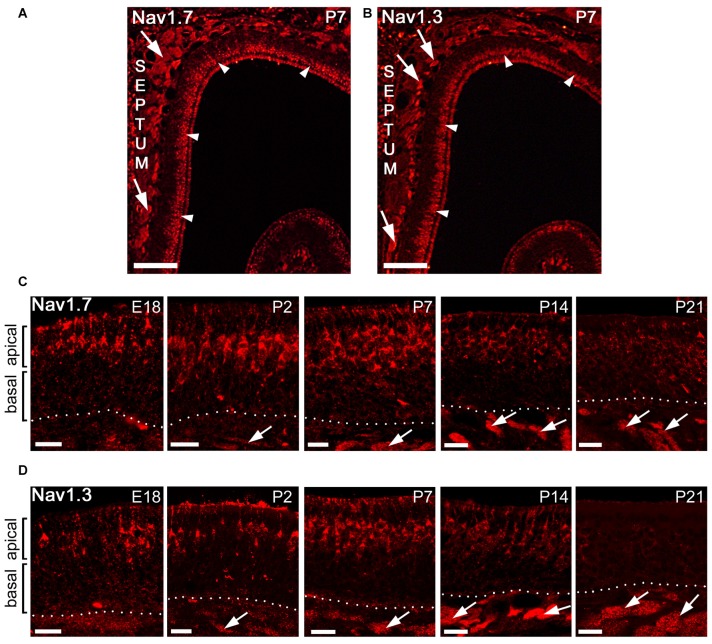
**Subcellular localization of Na_v_1.7 and Na_v_1.3 staining during mouse MOE development. (A,B)** Coronal tissue sections showing the dorsal aspect of the left nasal cavity (septum to the left) of a postnatal day 7 (P7) mouse. A strip of strong immunoreactivity is visible for **(A)** Na_v_1.7 and **(B)** Na_v_1.3 in the most apical MOE layer (arrowheads) and in axon bundles (arrows).** (C,D)** Higher magnifications of the MOE derived at different mouse ages stained with antibodies for** (C)** Na_v_1.7 and** (D)** Na_v_1.3. The confocal images show strongly stained OSN somata solely in the apical MOE. Somatic OSN staining is visible at embryonic day 18 (E18), P2, and P7, declines at about P14 and is nearly diminished at P21. Axon bundles stain early on (arrows) and increase in size with age in relation to epithelial thickness. Images are representatives of (*n* ≥ 2) mice at each age with *n* ≥ 10 sections per mouse. Scale bars **(A,B)** 200 μm, **(C,D)** 20 μm.

We then investigated the localization of Na_v_1.3 and Na_v_1.7 at different developmental time points in the MOE using immunohistochemistry. We analyzed MOE tissue sections derived from B6 or OMP-GFP mice at embryonic day 18 (E18), postnatal day 2 (P2), P7, P14 and P21 (Figures 4A–D). Surprisingly, between E18 and P7, we detected strong immunoreactivity for both Na_v_1.3 and Na_v_1.7 in OSNs that were primarily situated in the apical half of MOE (Figures 4C,D). The staining pattern at these ages was clearly different from that of adult tissue and showed robust labeling of OSN somata, dendrites and knobs (Figures 4C,D). The density of labeled OSN somata was relatively low at E18 but increased during development and reached its maximum at about P7, when immunoreactive OSN somata appeared tightly packed in the apical half of MOE (Figures 4C,D). At about P14, the somatic staining of OSNs started to disappear in the neuroepithelium lining the nasal septum and the dorsal roof, whereas olfactory turbinates maintained robust staining of somata at this age. At P21, the discerned staining for Na_v_1.3 and Na_v_1.7 in apical OSN somata diminished even in the olfactory turbinates (Figures 4C,D), and in adult mice pronounced somatic staining of OSN somata was absent. By contrast, immunoreactivity for Na_v_1.3 and Na_v_1.7 in OSN axon bundles was detectable at E18 and increased steadily with age and epithelial thickness (Figures 4C,D). Thus, the expression of Na_v_1.3 and Na_v_1.7 at somatic locations appears to be developmentally regulated and occurs in a transient manner, with a peak at P7, in contrast to the expression of these channels in OSN axons which happens steadily over developmental time.

### Enhanced Somatic Expression of Na_v_1.3 and Na_v_1.7 in Mature OSNs of Juvenile Mice

**Figure 5:**
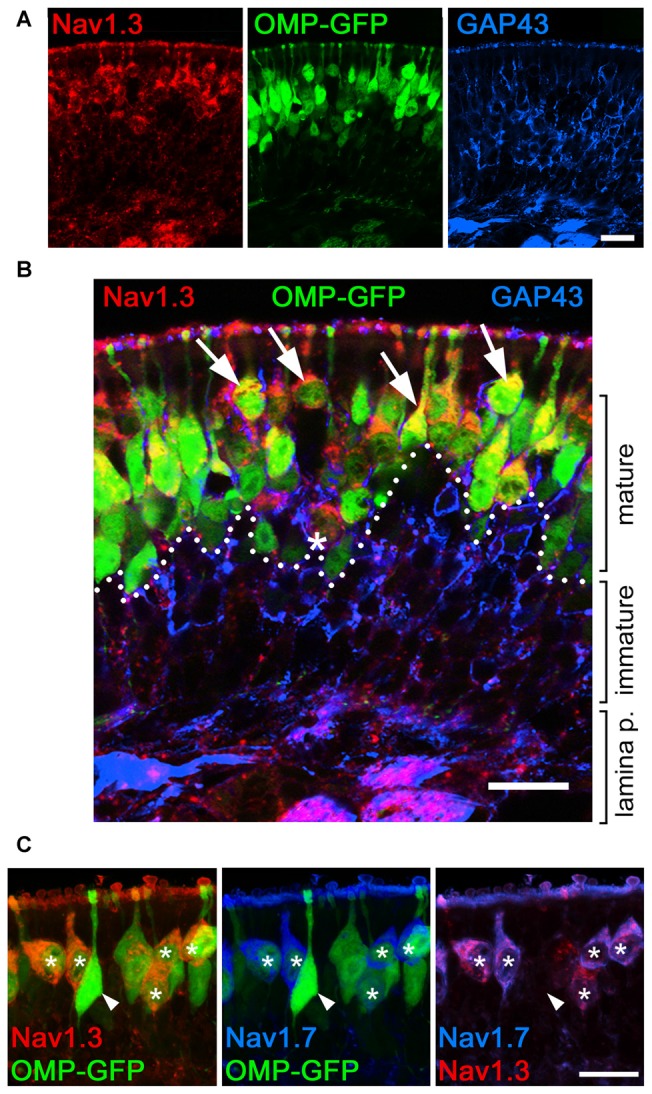
**Somatic expression of Na_v_1.3 and Na_v_1.7 occurs in mature OSNs.** Colocalization of Na_v_1.3, GAP43, and Na_v_1.7 in the olfactory epithelium of a P7 OMP-GFP mouse (endogenous fluorescence, green). **(A)** Single fluorescence images for Na_v_1.3 (red), OMP-GFP (green), and GAP43 (blue). **(B)** Magnified merge of the images in **(A)** shows that Na_v_1.3 colocalizes with OMP-GFP (arrows) in mature OSN somata located in the apical layer (dotted line). Occasionally, triple-labeled OSNs, positive for Na_v_1.3, OMP-GFP, and GAP43 were detected (asterisk). **(C)** Colocalization of Na_v_1.3 (red) and Na_v_1.7 (blue) in OMP-GFP^+^ OSNs (asterisks) intermingle with singly, OMP-GFP labeled OSNs (arrowhead). Images are representatives of (*n* = 2) mice with *n* ≥ 10 sections per mouse. Scale bars, 20 μm.

To gain further insight into the developmental regulation of Na_v_ channel expression at OSN somata, we performed additional experiments in early postnatal mice, at P7. First, we used OMP-GFP mice and conducted a 3D reconstruction of confocal Z-stacks to demonstrate that immunoreactivity for Na_v_1.3 and Na_v_1.7 in OSN somata colocalizes with endogenous GFP (Figures 5A–C). Thus, the transient expression of these two Na_v_ channels is confined to mature, OMP-GFP^+^ OSNs, although not all OMP-GFP^+^ OSNs were immunoreactive for either Na_v_1.3 or Na_v_1.7 (Figures 5B,C). Second, we assessed colocalization of the growth-associated protein GAP43 (Verhaagen et al., 1989), a marker for immature OSNs, with either Na_v_1.3 or Na_v_1.7 in OMP-GFP mice (Figures 5A,B). These experiments revealed that GAP43^+^ OSNs of the basal MOE lacked immunoreactivity for both channel subtypes, consistent with our result that the somatic expression of Na_v_1.3 or Na_v_1.7 occurs in mature OSNs. Occasionally, we detected a triple-labeled OSN cell body, positive for OMP-GFP, GAP43 and either Na_v_1.3 or Na_v_1.7, indicating that these cells comprised an early mature OSN phenotype (Figure 5B). Third, we assessed whether Na_v_1.3 and Na_v_1.7 are expressed in the same OSNs. We performed double-labeling experiments using antibodies raised in different species: a Na_v_1.3 antibody raised in goat and a rabbit antiserum for Na_v_1.7. As illustrated by the confocal images depicted in Figure 5C, both Na_v_1.3 and Na_v_1.7 colocalize in the same OSN somata.

### Plasticity and Restoration of Na_v_1.3 and Na_v_1.7 Expression in the Regenerating MOE

Mammalian OSNs have the capacity to turn-over and regenerate throughout the animal’s life span (Graziadei, G. A. and Graziadei, P. P. C., 1979; Graziadei, P. P. C. and Graziadei, G. A., 1979). Having shown that transient expression of Na_v_1.3 and Na_v_1.7 at OSN cell bodies coincides with a critical period of heightened plasticity during early postnatal maturation (Figures 4, 5), we next asked whether somatic expression of Na_v_1.3 and Na_v_1.7 can also be induced during regeneration and *de novo* OSN synthesis in the MOE. To test this, we performed peripheral deafferentation of the MOE through intranasal application of Triton X-100, an established method which induces severe chemical lesion of the neuroepithelium that is followed by tissue degeneration and subsequent regeneration from a pool of mitotic active stem cells located at the basal membrane (Nadi et al., 1981; Verhaagen et al., 1990).

**Figure 6:**
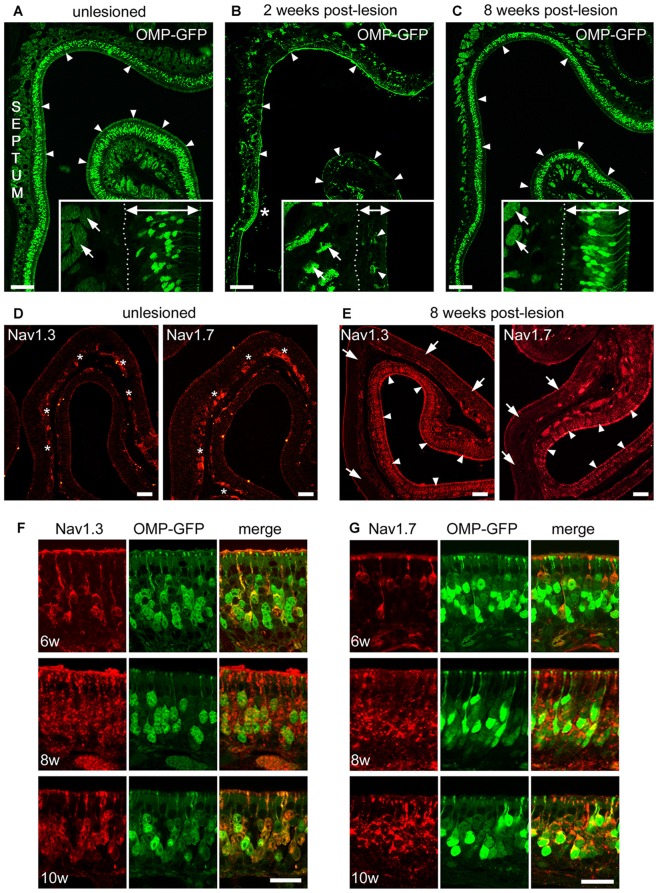
**Recovery of Na_v_1.3 and Na_v_1.7 expression in OSN somata during regeneration from chemical ablation. (A–C)** Coronal MOE sections of the anterior left nasal cavity (septum to the left) of OMP-GFP mice. **(A)** Untreated control mice display OMP-GFP labeling of OSNs throughout the MOE (arrowheads). Inset magnification exemplifies regular thickness of axon bundles (arrows) and the MOE (double arrow, 80 μm). **(B)** Severely damaged MOE 2 weeks post-lesion. Small patch of unlesioned MOE at the septal wall (asterisk). The inset magnification depicts the reduced thickness of axon bundles (arrows) and the MOE (double arrow, 35 μm) 2 weeks post-lesion. Few newly generated OMP-GFP positive OSNs are visible (arrowheads). **(C)** The MOE has largely recovered 8 weeks post-lesion (arrowheads). The inset magnification shows that the thickness of axon bundles (arrows) and the MOE (double arrow, 70 μm) is increased. Basal membrane (dotted line). **(D)** Immunoreactivity for Na_v_1.3 and Na_v_1.7 in the intact, unlesioned MOE shows strong labeling of axon bundles (asterisks). **(E)** Eight weeks post-lesion, tissue stretches with heavy immunolabeling for Na_v_1.3 and Na_v_1.7 in OSN somata (arrowheads) reside side-by-side with areas devoid of any somatic immunoreactivity (arrows). This pattern likely coincides with the different levels of initial damage yielding various levels of MOE regeneration.** (F,G)** Magnifications of the MOE at 6, 8, and 10 weeks post-lesion showing somatic staining for **(F)** Na_v_1.3 (red) and **(G)** Na_v_1.7 (red) colocalizing with OMP-GFP (green). Images are representatives of (*n* = 2) mice at each recovery time point with *n* ≥ 20 sections per mouse. Scale bars **(A–C)** 200 μm, **(D,E)** 50 μm **(F,G)** 20 μm.

We performed Triton X lesions using adult, 8-week-old OMP-GFP mice and analyzed regeneration of the MOE over a time course of 1, 2, 4, 6, 8 and 10 weeks, respectively. One week after lesioning, the MOE was still severely damaged. Two weeks after lesion, the thickness of the MOE was recovered to about one third compared to unlesioned control tissue. At that time, we observed the first newly generated OMP-GFP^+^ OSNs (Figures 6A,B) that were sparsely distributed in the regenerating MOE (Figures 6A,B). Small patches of epithelium remained intact in areas where lesions had been incomplete (Figure 6B). However, axon bundles in the underlying lamina propria had shrunk remarkably in the whole epithelium. Over the following weeks of recovery, the number of OMP-GFP^+^ OSNs increased continuously and the epithelial thickness reached ~90% of control levels by 8 weeks post-lesion (Figure 6C).

With respect to the expression of Na_v_ channels during this regeneration process, at about 4 weeks post-lesion we occasionally detected immunostaining for Na_v_1.3 and Na_v_1.7 in single OSN somata, however, staining intensity was close to the detection threshold. At about 6 weeks post-lesion, immunoreactivity for both Na_v_ channels was evident in a substantial number of OSNs exhibiting robust labeling of somata, dendrites, and dendritic knobs (Figures 6E–G). The density of labeled OSNs increased towards 8 weeks post-lesion and robust somatic staining was still evident at 10 weeks post-lesion. Colocalization with OMP-GFP showed that Na_v_-positive OSNs also expressed GFP. Overall, the expression pattern closely resembled that of P7 mice during early postnatal development (for comparison, see Figure 4C). Thus, the expression of Na_v_1.3 and Na_v_1.7 in OSN somata appears to be highly plastic and seems to be linked to the establishment of the neuroepithelium during ontogeny and during regeneration following Triton X lesioning.

## Discussion

We have addressed the molecular basis of neuronal excitation in the mammalian olfactory system by investigating the organization and plasticity of Na_v_ channel expression in the peripheral olfactory system of mice during adulthood and development of the MOE and VNO, and during regeneration of the MOE following chemical epithelial ablation. We used a combination of qPCR and immunohistochemistry to reveal fundamental similarities, but also important differences in the Na_v_ repertoire employed by the two major olfactory subsystems, the MOE and the VNO. (1) PCR results show that Na_v_1.7 is the predominant isoform not only in the MOE but also in the VNO. (2) We provide immunohistochemical evidence that both Na_v_1.3 and Na_v_1.7 maybe fundamental for propagating action potentials in the two olfactory subsystems as they are primarily located to axons of sensory neurons. (3) We also show for the first time the complex organization of Na_v_ channel expression in the VNO, which involves at least four different subtypes with different biophysical properties. In addition to Na_v_1.3 and Na_v_1.7 in VSN axons, we find robust expression of Na_v_1.2 and Na_v_1.6 in VSN somata. Na_v_1.2 only exists in VSNs located in the basal VNO layer and is expected to have a specific function in these neurons. (4) Finally, we show that Na_v_1.3 and Na_v_1.7 undergo changes in subcellular localization during the first weeks of development and during regeneration of the MOE following chemical lesion, which likely reflects specific physiological requirements associated with neuronal activity during periods of heightened plasticity.

### Na_v_ Channel mRNA Expression in MOE and VNO

**Figure 7:**
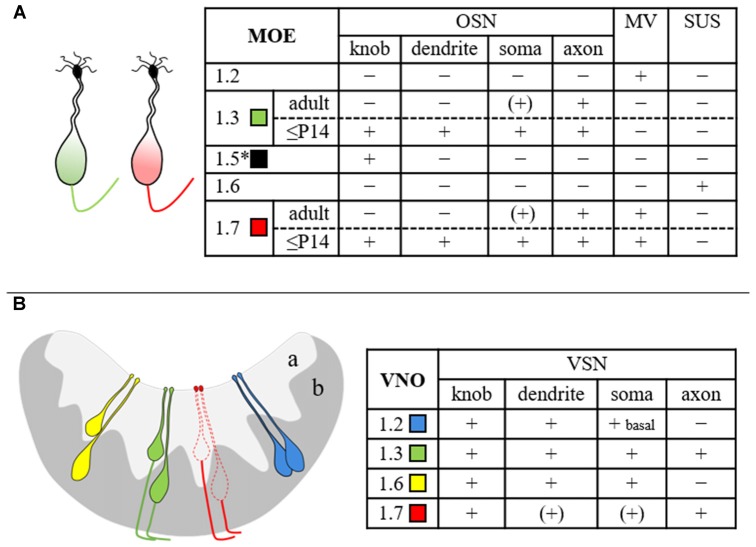
**Summary scheme depicting the cellular and subcellular distribution of various Na_v_ channel isoforms in mouse MOE (A)** and VNO **(B)**. The drawings at the left illustrate the results obtained in this study and refer to the table at the right. Expression of Na_v_1.5 (asterisk) in OSN dendritic knobs (Frenz et al., 2014) has been included for completeness. The different Na_v_ channel subtypes are color-coded as indicated. The extent of immunoreactivity was categorized as + (present), − (absent), or (+) (close to detection threshold). MV, microvillar cells; OSN, olfactory sensory neuron; SUS, sustentacular cells; VSN, vomeronasal sensory neuron; a, apical VNO layer; b, basal VNO layer.

To address the question whether the MOE and the VNO share the same Na_v_ channels or whether each system employs a unique set of channels, we first compared the expression profiles in the two tissues. Using real-time quantitative PCR, we identified six different Na_v_ channel mRNAs in both MOE and VNO, demonstrating that these tissues share the same channels. Our results in the MOE are supported by previous reports that have identified these isoforms by RT-PCR (Weiss et al., 2011; Frenz et al., 2014) and deep RNA sequencing (Ibarra-Soria et al., 2014). In the VNO, Fieni et al. (2003) identified mRNAs encoding Na_v_1.1, Na_v_1.2, and Na_v_1.3. Our study confirms these isoforms and extends the VNO repertoire by the isoforms Na_v_1.5, Na_v_1.6, and Na_v_1.7. The frequency of individual isoforms was similar between the two olfactory subsystems, with Na_v_1.7 being the predominant isoform in the MOE and the VNO. These results are also consistent with the quantitative results from deep RNA sequencing (Ibarra-Soria et al., 2014). Despite these comparable mRNA repertoires, subsequent immunohistochemical localization analyses revealed specific differences and showed that the cellular and subcellular protein distribution of Na_v_ channels is not identical in the two olfactory tissues (for summary see Figure 7).

### Sensory Neurons of the MOE Express Na_v_1.3 and Na_v_1.7

In the MOE, we detected robust expression of Na_v_1.3 and Na_v_1.7 in OSN axons and substantially lower levels in OSN somata. This is consistent with previous results detecting both isoforms in OSN axons coursing the nerve layer of the main olfactory bulbs (Weiss et al., 2011). It was shown that Na_v_1.7 but not Na_v_1.3 is expressed in the axon terminals of OSNs, and that the unique localization of Na_v_1.7 may be critical for the synaptic transmission of olfactory information to the main olfactory bulb (Weiss et al., 2011; Zufall et al., 2012). A similar subcellular distribution has been shown for Na_v_1.7 in rat (Ahn et al., 2011). The redundancy of Na_v_1.3 and Na_v_1.7 in OSN axons implies that the two channel subtypes likely function in a coordinated manner in the generation and propagation of action potentials. Na_v_1.3 and Na_v_1.7 display similar biophysical properties, including fast activation and inactivation kinetics and a slow closed-state inactivation that enables inward currents even in response to weak depolarizations (Klugbauer et al., 1995; Cummins et al., 2001; Herzog et al., 2003). However, Na_v_1.3 recovers three times faster from inactivation than Na_v_1.7 (Cummins et al., 2001). It is therefore conceivable that Na_v_1.3 bypasses the longer recovery times of Na_v_1.7 to maintain firing at high frequency after odor-induced depolarization.

We did not detect Na_v_1.3 or Na_v_1.7 in dendritic endings of OSNs. This location is occupied by Na_v_1.5, a subtype that has been shown recently to contribute to spontaneous OSN activity (Frenz et al., 2014) and may transduce small, odorant-evoked receptor potentials into action potential firing (Dionne, 2016). Together, these results suggest that OSNs employ at least three different Na_v_ channel subtypes for action potential generation, propagation, and signal transmission—Na_v_1.5 in dendritic knobs, Na_v_1.3 and Na_v_1.7 in somata and axons, and Na_v_1.7 in axon terminals of OSNs.

### Sensory Neurons of the VNO Employ Four Different Na_v_ Channel Subtypes

Only limited information has been available on the molecular identity of the Na_v_ channel subtypes employed by the VNO. Thus far, only one study has demonstrated expression of the subtype Na_v_1.3 in VNO tissue sections using *in situ* hybridization (Fieni et al., 2003). We now demonstrate that at least four Na_v_ channel subtypes with different biophysical properties are expressed in sensory neurons of the VNO. The four channels exhibit differential but not exclusive expression in specific subcellular VSN compartments and display onset of expression during the first postnatal week of life (Figure 3). Consistent with their presumptive function in action potential generation and impulse propagation in the vomeronasal system, we identified Na_v_1.2, Na_v_1.3, Na_v_1.6, and low levels of Na_v_1.7 in somata and Na_v_1.3 and Na_v_1.7 in axons of VSNs (Figure 3), respectively. Na_v_1.6 expression was stronger in VSN somata of the basal VNO layer than in the apical layer, whereas Na_v_1.3 was uniformly expressed in all VSNs. Most intriguingly, Na_v_1.2 was limited to VSN somata of the basal layer, suggesting that this channel may play a special role in these VSNs. This is in agreement with earlier reports demonstrating that the electrophysiological characteristics of sodium currents differ in apical vs. basal VSNs (Liman and Corey, 1996; Fieni et al., 2003; Ukhanov et al., 2007; Ackels et al., 2014). Na_v_ currents of basal VSNs were shown to be smaller using a dissociated VNO preparation (Fieni et al., 2003). Using an intact VNO slice preparation and genetically-identified VSNs, VSNs of the basal VNO layer were shown to exhibit larger currents and to produce faster and larger spikes than apical VSNs (Ukhanov et al., 2007). Interestingly, Na_v_1.2 is characterized by fast inactivation kinetics and by generating repetitive action potential firing (Catterall et al., 2005), which could explain that basal VSNs are capable to maintain persistent firing for extended periods of time (Ukhanov et al., 2007). Thus, the coordinated action of three different Na_v_ channels in basal VSNs and two different Na_v_ channels in apical VSNs enable the finely-tuned control of action potential firing in the respective VNO layers.

Furthermore, the robust expression of Na_v_1.3 and Na_v_1.7 we observed in VSN axons parallels our results in the MOE and suggests an analogous function of these Na_v_ channels in the accessory olfactory system. We propose that Na_v_1.3 and Na_v_1.7 represent fundamental subtypes in the conduction of electrical signaling in both VSNs and OSNs. Moreover, consistent with the recent observation that Na_v_1.7 is expressed in the nerve and glomerular layers of the accessory olfactory bulb in rat (Rupasinghe et al., 2012) and our unpublished results in mouse, we suggest that similar to the MOE (Weiss et al., 2011), Na_v_1.7 may play an essential role in olfactory signal transmission at the first synapse of the AOB.

### Special Roles for Na_v_1.3 and Na_v_1.7 during MOE Development and Regeneration

The generation of neural activity through action potentials is a major determinant in the regulation of development and plasticity of the nervous system (Hensch, 2004; Holtmaat and Svoboda, 2009). Having shown that Na_v_1.3 and Na_v_1.7 represent fundamental Na_v_ channels for action potential propagation in the adult, we focused on the developmental expression of the two Na_v_ channels in the MOE. Our results show that the subcellular distribution of Na_v_ channels in young mice was remarkably different from adults. Between E18 and P14, expression of Na_v_1.3 and Na_v_1.7 was pronounced in somata, dendrites, and knobs of OSNs and was limited to mature, OMP-positive OSNs. Somatic expression peaked at about P7 and subsided within the following 2 weeks to the low levels observed in the adult, while axonal expression was maintained from E18 on. This differential, time-dependent expression of Na_v_ channels in OSN compartments coincides with a critical period of heightened plasticity during early postnatal life (Hensch, 2004) and is likely to meet specific requirements associated with increased neuronal activity. Electrical activity promotes axon outgrowth (Mobley et al., 2010), olfactory synapse formation (Cheetham et al., 2016), and establishment of a topographic map in the olfactory bulb (Yu et al., 2004; Ma et al., 2014). Furthermore, silencing spontaneous activity delays axonal outgrowth (Mobley et al., 2010) and precise axonal targeting to specific glomeruli in the olfactory bulb (Yu et al., 2004; Ma et al., 2014; Tsai and Barnea, 2014). Interestingly, the time course of somatic Na_v_ expression we observed in this study coincides with that of olfactory synapse maturation and refinement. Synapse formation between OSNs and postsynaptic mitral/tufted cells in the main olfactory bulbs starts at about embryonic day 15 and continues throughout life (Hinds and Hinds, 1976; Blanchart et al., 2008). Exuberant axonal projections and synapses peak at about postnatal day 8 and are eliminated towards postnatal day 20 (Marcucci et al., 2011). Thus, it is conceivable that Na_v_-dependent electrical activity may contribute to refinement or strengthening of olfactory synapses formed during early postnatal development. Furthermore, we showed that expression of Na_v_1.3 and Na_v_1.7 in OSN somata was confined to mature, OMP-positive somata (Figure 5), which is consistent with the observation that the onset of OMP expression closely associates with synapse formation (Graziadei et al., 1978; Farbman and Margolis, 1980; Rodriguez-Gil et al., 2015; Cheetham et al., 2016). Thus, somatic Na_v_ expression in OSNs is turned-off following synapse refinement whereas axonal expression of Na_v_ channels is maintained. However, at the current state we cannot exclude the possibility that somatic Na_v_ expression is linked to other developmental processes, as well.

In contrast to embryonic and early postnatal mice, we never detected such striking Na_v_ immunoreactivity in OSN somata of the adult MOE, as might be expected from OSNs that emerge during adult neurogenesis. This observation is in line with the idea that mechanisms regulating embryonic, juvenile, and adult neurogenesis are overlapping but not identical (Brann and Firestein, 2014; Ma et al., 2014). Adult born OSNs differentiate in the context of an established olfactory network in which somatic Na_v_ expression may be dispensable. To test whether the transient expression of Na_v_ channels in OSN somata can be restored during regeneration of the MOE, we used Triton X to reversibly lesion the MOE and to track Na_v_ channel expression during *de novo* synthesis of OSNs. Unlike other sensory systems, the olfactory system has a remarkable regenerative capacity due to a pool of stem cells located at the basal lamina of the MOE (Graziadei, G. A. and Graziadei, P. P. C., 1979; Graziadei, P. P. C. and Graziadei, G. A., 1979). Consistent with earlier reports (Nadi et al., 1981; Verhaagen et al., 1990), our results show that after massive degeneration of the MOE during the first week post-lesion, newly generated, OMP-positive OSNs emerge within 2 weeks post-lesion. About 6 weeks post-lesion, we detected robust immunostaining for Na_v_1.3 and Na_v_1.7 in OSN somata (Figure 6), which was similar to the expression pattern we observed during late embryonic and early postnatal MOE development.

Thus, the somatic expression of Na_v_1.3 and Na_v_1.7 in OSNs appears to be highly plastic which is consistent with specific roles for the two Na_v_ channels. Although lesion-evoked MOE regeneration in adult mice is not simply a recapitulation of ontogeny, our data suggest that somatic expression of Na_v_1.3 and Na_v_1.7 might be part of a program involving increased electrical activity during the initial maturation process and during recovery from injury of the olfactory epithelium.

In summary, we have provided a systematic analysis of the expression of Na_v_ channel isoforms in the peripheral mammalian olfactory system. These experiments reveal complex patterns and highly specific differences of Na_v_ channel expression between the MOE and VNO, and during periods of high plasticity of these tissues. Experiments using conditional mutations in these Na_v_ channel subunits, akin to those used for understanding the role of Na_v_1.7 (Weiss et al., 2011), will be required to analyze the precise functional contribution of each these ion channels to olfactory performance.

## Author Contributions

MP: study concept; FB, SK and MP: data acquisition (immunohistochemistry, confocal microscopy; FB (Triton-x lesion), BB (qPCR)). FB, BB, SK, MP and FZ: data interpretation; MP: figure preparation; MP and FZ: drafting of the manuscript; FB, BB: revision of the manuscript. All authors take responsibility for data integrity and data analysis accuracy.

## Funding

This work was supported by Deutsche Forschungsgemeinschaft (DFG) grants PY 90/1-1 (to MP) and SFB 894 (to FZ).

## Conflict of Interest Statement

The authors declare that the research was conducted in the absence of any commercial or financial relationships that could be construed as a potential conflict of interest. The handling Editor declared a past co-authorship with one of the authors MP and the handling Editor states that the process met the standards of a fair and objective review.
